# Standardisation of high throughput microdilution antifungal susceptibility testing for *Candida albicans* and *Cryptococcus neoformans*

**DOI:** 10.1038/s41598-024-74068-2

**Published:** 2024-10-08

**Authors:** Holly E. E. Floyd, Angela M. Kavanagh, Gabrielle J. Lowe, Maite Amado, James A. Fraser, Mark A. T. Blaskovich, Alysha G. Elliott, Johannes Zuegg

**Affiliations:** 1 Community for Open Antimicrobial Drug Discovery, Centre for Superbug Solutions, Institute for Molecular Bioscience, The University of Queensland, Brisbane, QLD Australia; 2 School of Chemistry and Molecular Biosciences, The University of Queensland, Brisbane, QLD Australia

**Keywords:** M27 guidelines, Broth microdilution, Susceptibility testing, Minimum inhibitory concentration, High throughput screening, Antifungal, *Candida albicans*, *Cryptococcus neoformans*, Antimicrobials, Fungi, High-throughput screening, Phenotypic screening

## Abstract

The Clinical and Laboratory Standards Institute (CLSI) M27 guidelines are the recommended and most commonly used protocols for broth microdilution antifungal susceptibility testing of yeasts. However, these guidelines are limited to the use of 96-well assay plates, limiting assay capacity. With the increased risk of fungal resistance emerging in the community, it is important to have alternative protocols available, that offer higher throughput and can screen more than eight to ten potential antifungal compounds per plate. This study presents an optimised broth microdilution minimum inhibitory concentration (MIC) method for testing the susceptibility of yeasts in an efficient high throughput screening setup, with minimal growth variability and maximum reproducibility. We extend the M27 guidelines and optimise the conditions for 384-well plates. Validation of the assay was performed with ten clinically used antifungals (fluconazole, amphotericin B, 5-fluorocytosine, posaconazole, voriconazole, ketoconazole, itraconazole, caspofungin diacetate, anidulafungin and micafungin) against *Candida albicans* and *Cryptococcus neoformans*.

**Supplementary Information:**

The online version contains supplementary material available at 10.1038/s41598-024-74068-2.

## Introduction

Resistant fungal infections have increased considerably in the last decade, leading to the World Health Organisation (WHO) to issue a fungal priority pathogens list to guide research, development and public health action^1^. They are caused by nosocomial organisms and opportunist pathogens. *Candida albicans* is the most prevalent fungal species present in the human microbiota, it colonises mucosal surfaces, such as the gastrointestinal tract, oral cavity, reproductive tract and skin of healthy adults^2^. However, variations in the host immune response or resident microbiota can enable *C. albicans* to overgrow and cause infection^3^. *C. albicans* is recognised as one of the most frequent human pathogens discovered in immunocompromised patients, as a consequence of advanced age, infection or immunosuppressive therapy^4,5^, and is one of four pathogens in the ‘critical priority group’ in the 2022 WHO fungal report. In addition to *C. albicans*, other species such as *Candida glabrata*,* Candida tropicalis* (WHO ‘high priority’), *Candida parapsilosis* (WHO ‘high priority’), *Candida krusei* and *Candida auris* (WHO ‘critical priority’) can cause debilitating and recurring infections in these mucosal surface areas^5,6^. In recent years, there has been an increase of emerging *Candida* infections reported worldwide, caused by species such as *Candida guilliermondii*, *Candida kefyr*, *Candida rugosa*, *Candida dubliniensis*, and *Candida famata*^7^. The National Nosocomial Infections Surveillance System (NNISS) has reported *Candida* species as one of the most common nosocomial bloodstream pathogen with a high estimated mortality rate (45%), with the high mortality most likely due to ineffective diagnostic techniques, inappropriate antifungal treatment^7^ and increasing antifungal resistance.

*Cryptococcus neoformans* (WHO ‘critical priority’) is an environmental microbe that lives in a yeast-like form, and is the pathogen responsible of causing life-threatening infections like cryptococcosis and meningoencephalitis. People are infected with *C. neoformans* from the environment, most likely by inhalation of infectious particles^8^. In healthy hosts, the primary infections can be asymptomatic. Serious illness arises in cases of deficiency or absence of intact cell-mediated immunity, and typically infections are found in immunocompromised patients^9^, people undergoing prolonged treatment with corticosteroids^10^ or immunosuppressive therapies and patients with advanced AIDS^11,12^. Some infections can be caused by reactivation of latent infection. *C. neoformans* has well-defined virulence factors, as well as the capability to grow at 37°C, to form a polysaccharide capsule and to produce spores^13^. It is also able to “replicate intracellularly and can escape the intracellular state without being killed or killing the host cell”^9^. Cryptococcus is the most common cause of meningitis in adults living with HIV in sub-Saharan Africa, with an estimated 181,000 deaths globally in 2014^12^, representing 15% of AIDS related deaths due to cryptococcal meningitis.

*C. albicans* and *C. neoformans* have developed a set of strategies to survive in hostile hosts. Antifungal resistance is based on adaptation mechanisms in the presence of toxic drugs, and include: mutations of drug targets reducing affinity for the drug, overexpression of the targeted protein, expression of an efflux system, degradation of the drugs and finally pleiotropic drug responses^7,14^. The treatment for infections caused by *C. albicans* and *C. neoformans* predominantly relies on a single class of antifungals known as azoles. The worldwide deployment of azoles, combined with the fungistatic nature of these drugs has contributed to the rise of azole resistance in clinical isolates^14^. Fluconazole resistant *C. neoformans* strains have been the most common, arising from AIDS patients undergoing long-term azole therapy^10^. The clinical relevance of these strains has contributed to the urgency to find new antifungals that can target these strains.

Drug discovery projects are often initiated with a high throughput screening campaign, testing hundreds of thousands to millions of chemically diverse compounds. In order to increase the number of antifungals available in the clinic, it is important to improve the throughput of Antifungal Susceptibility Testing (AST) available^15^ and scale testing to a high throughput screening format to increase the chances of finding more efficient and diverse therapies. The guidelines of the Clinical Laboratory Standards Institute (CLSI) describe methods to measure antimicrobial susceptibility for *Candida* and *Cryptococcus spp* in 96 well plates^16^, suggesting Roswell Park Memorial Institute (RPMI) 1640 media with MOPS (3-morpholinopropane-1-sulfonic acid) buffer incubated at 35 °C for 48 h for *C. albicans* and 72 h for *C. neoformans*. However, other committees such as the European Committee on Antimicrobial Susceptibility Testing (EUCAST)^17^ and several publications have proposed different conditions. The suggestions include different conditions for temperature (30 °C and 35 °C), agitation and static conditions, different inoculum preparation and initial inoculum density (10^3^, 10^4^, and 10^5^ CFU/mL)^15^, alternative media^17^, and different incubation times^18^. It is worth noting that both CLSI and EUCAST provide guidelines which are primarily aimed to test the susceptibility of new clinical isolates against known antimicrobials, and not so much for testing novel compounds against known isolates, in a high throughput screening approach in which generally tests single concentration for primary screening and dose response testing only for confirmational screening.

In this study we compared conditions from the CLSI guidelines with conditions described in the literature, such as alternative temperature, media and incubation time, and we applied them to a 384 well plate format for high throughput screening. The study was able to select optimal conditions for the screening against *C. albicans* and *C. neoformans*, in 384 well plates with absorbance readout, as measured by optimal growth and by minimum inhibitory concentration (MIC) profile against a panel of antifungals.

## Results

### Growth curves in 384 well plates

Both strains, *C. albicans* (ATCC 90028) and *C. neoformans* (ATCC 208821), were suspended and diluted to a final concentration of 2.5 × 10^3^ CFU/mL in fresh Yeast Nitrogen Base (YNB) broth and RPMI-1640 with MOPS Medium. The suspension was added to polystyrene (PS) and non-binding surface polystyrene (NBS) 384 well plates and placed in an incubator at 30 °C and 35 °C. The growth of the yeast was thereby measured in two ways, by measuring the turbidity of each well by an absorbance at a wavelength of 630 nm (OD_630_), and by taking samples from the wells and counting the colony forming units (CFU) on Yeast Extract-Peptone Dextrose (YPD) agar plates. Both measurements were taken at 0, 18, 24, 36, 48 and 72 h time points.

The CFU/mL counts displayed very little differences for both fungal species, when using either YNB or RPMI as growth media. In contrast, the OD_630_ readouts using the same growth conditions exhibited substantial differences between the two media. For both species, using the YNB media resulted in higher OD_630_ reads compared to RPMI.

Spectrometric turbidity measurement is a fast and simple surrogate method to measure antimicrobial growth, allowing the scale up of growth inhibition assays to be conducted as high throughput screening in 96 or 384 well microtiter plates without the need for manual growth/no-growth assessment. The method is, however, dependent on the specific assay setup, including labware, final test volume and absorbance reader. To exclude possible effects from the labware, all assays were repeated in standard polystyrene (PS) 384-well plates (Appendix Figs. 1 and 2), giving similar results as when using the NBS 384-well plates.

A further limitation of measuring antimicrobial growth by absorbance is that, the linearity of turbidity is limited to a narrow range of microbial CFU. For bacteria such as *E. coli*, OD_600_ values of 0.1 and 1.0 correspond to a narrow range of 10^8^ and 10^9^ CFU/mL, with small variations between different experimental setup. Similarly, for fungi such as *C. albicans*, OD_630_ values of 0.1 and 1.0 correspond to 10^7^ and 10^8.5^ CFU/mL^19^. In this sense, the two-fold difference in OD_630_ values seen between the YNB and RPMI media would correspond to a CFU/mL difference equivalent to 2 × 10^7^ to 4 × 10^7^, which is close to the experimental error of the CFU measurement. In addition, different media and plate surfaces will also affect the morphology of the fungi, leading to different turbidity values at similar CFU concentrations^20,21^.

In the context of high throughput screening for growth inhibition, the quality of the screen is not such much defined by the absolute measurement of CFU/mL, but by the separation between growth (> 10^7^ CFU/mL) and no growth (< 10^3^–10^5^ CFU/mL, depending on the limit of detection). The Z’-factor is thereby used as a quality control of the assay performance, calculated as the ratio between 3 times the sum of variation of positive and negative controls, and the difference between positive and negative controls^22^.

## **Edge effect in 384 well plates**

**Fig. 1:**
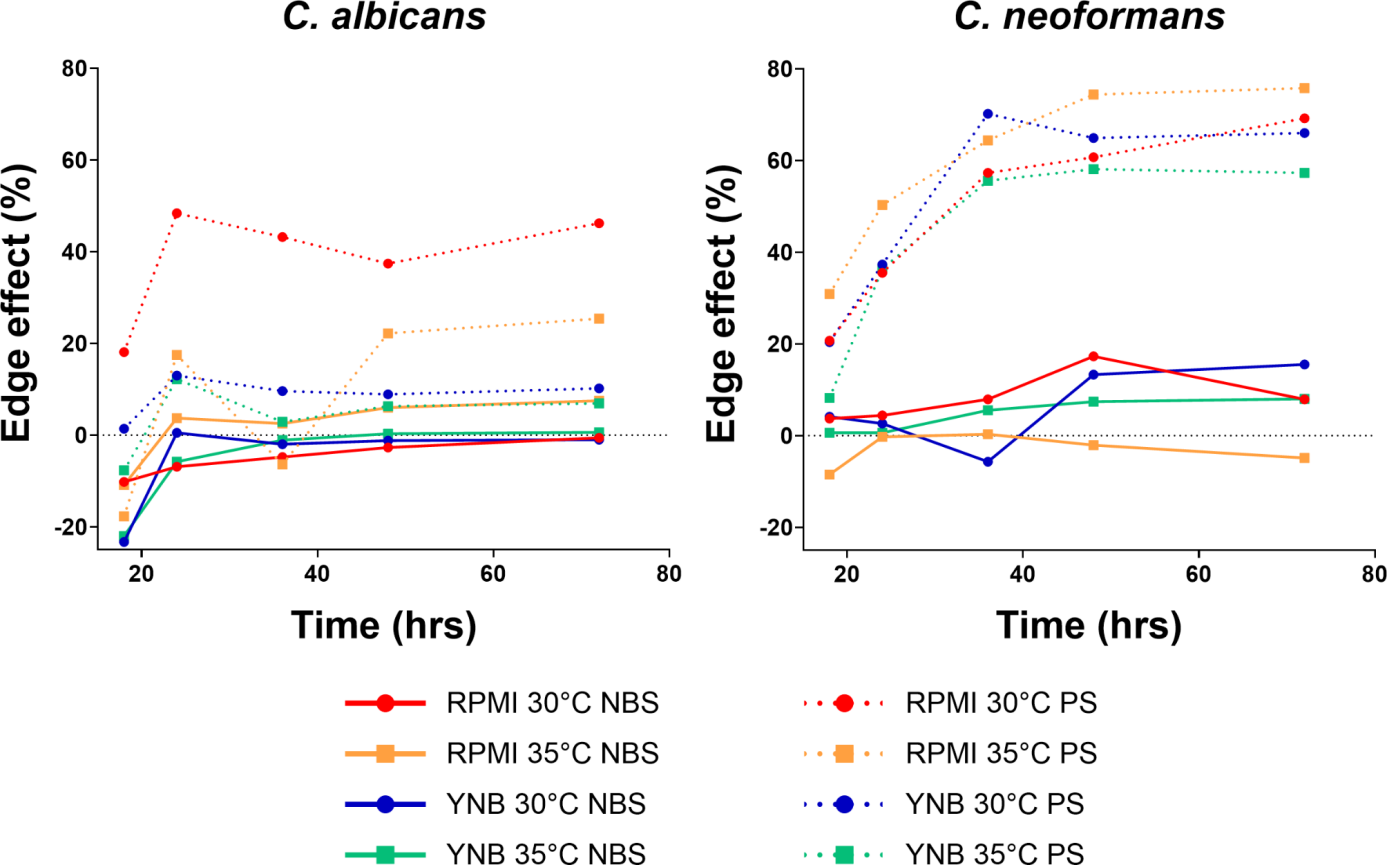
Edge effects at different growth times, for *C. albicans* and *C. neoformans*, using RPMI and YNB media, at 30 °C and 35 °C, and using NBS and PS plates.

As 384-well plates have an elevated risk to introduce edge effects in growth due to reduced well volumes (100 µL in standard 96 well plate, 50 µL in 384 well plate), we monitored the edge effect from the growth curve experiments, by calculating the average percentage OD_630_ values of the edge wells compared to the wells in the middle (Appendix Formula 1 and 2). As Fig. 1 shows, the edge effect was more prominent for *C. neoformans* than *C. albicans*. But more surprisingly, the edge effect was mainly dependent on the plate type. Even though the overall growth was similar between the plate types, the PS plates displayed a noticeably higher edge effect for both species. The non-binding surface (NBS) plates displayed a low average edge effect of -3.6% and 3.9% for *C. albicans* and *C. neoformans* respectively. These edge effects are within the variations of the wells in the middle, with average Z-scores for the edge wells at 0.38 and 0.42, for *C. albicans* and *C. neoformans* respectively (Appendix Table 2). In comparison, when using PS plates the average edge effect was much higher, with 14.9% (Z-Score: 0.71) and 50.7% (Z-Score: 2.71) of additional growth for *C. albicans* and *C. neoformans* respectively. For *C. neoformans* these edge wells lay outside the 99% confidence interval and are classified as significant outliers.

For high throughput screening against *C. neoformans* using the NBS plate would provide a significant performance improvement for either media, while against *C. albicans* NBS plates would be preferred over PS plates, as long as YNB media is used.

## **Antifungal susceptibility testing in 384-well plates**

The CLSI standard for antifungal susceptibility test has facilitated the establishment of minimum inhibitory concentration (MIC) breakpoints for a number of antifungals, such as fluconazole, amphotericin, itraconazole, voriconazole, and against a number of *Candida*spp sppspecies. To validate our high throughput method for the measurement of MIC values, we conducted dose response screens of several antifungals against both strains, using YNB and RPMI at 35 °C, and using different incubation times (36, 48 and 72 h).

After incubation, optical density was measured at 630 nm (OD_630_) for *C. albicans* growth. For *C. neoformans* we initially measured OD_630_, which however resulted in poor assay performance parameters with Z’-factor at an average of only 0.32. To improve the assay performance we conducted the assay with the addition of resazurin (final concentration of 0.01%) after incubation and the measurement of the difference in optical density between 570 and 600 nm (OD_570 − 600_) resulting in an improved average Z’-factor of 0.64. Any reported MIC assay against *C. neoformans* in YNB was conducted with the addition of resazurin (Table 1). We determined the MIC for each antifungal compound by calculating the percentage of inhibition of each well using media only as negative (100% inhibition) and fungi only as positive control (0% inhibition), and classifying active from non-active wells by using a threshold of 80% inhibition (Tables 1 and 2). For MIC_2_ a threshold of 50% inhibition was used (Appendix Tables 3 and 5) to represent the half activity equivalent to CLSI score 2.

**Table 1:** MIC (µg/mL) of tested antifungals against *C. Albicans* ATCC 90028 in 384-well non-binding surface (NBS) plates, by OD_630_ readout (*n* = 4). # amphotericin B prepared from solution stock.

Antifungal	Inhibition of C. albicans ATCC 90028		
	MIC (µg/mL)		
	RPMI 48 h	YNB 36 h	CLSI or Literature
Ketoconazole	0.004	0.031	0.031^28^
Posaconazole	0.001	≤ 0.0005	-
Itraconazole	0.004–0.016	0.002–0.004	0.016^28^
Fluconazole	2	0.5	0.25–1^24^
Voriconazole	0.002–0.004	0.002	0.004-0.016^29^
Anidulafungin	≤ 0.0005	0.016	0.03
Caspofungin	≤ 0.0005	≤ 0.0005	0.125^30^
Micafungin	0.001	0.008	0.016^31^
Amphotericin B	0.125, 0.125^#^	0.125, 0.25^#^	0.5–2^24^
5-Flucytosine	0.5-2	0.125	0.5–2^24^

**Table 2:** MIC (µg/mL) of tested antifungals against *C. Neoformans* var. *Grubii* (H99) ATCC 208821 in 384-well NBS plates, by OD_570 − 600_ readout with resazurin (*n* = 4). # amphotericin B prepared from solution stock, * literature MICs for 5-flucytosine against *C. neoformans* are not strain specific.

Antifungal	Inhibition of C. neoformans ATCC 208821		
	MIC (µg/mL)		
	RPMI 48 h	YNB 36 h	CLSI or Literature
Ketoconazole	0.031	0.5-2	0.06^32^
Posaconazole	≤ 0.0005–0.008	0.03	0.063^33^
Itraconazole	0.031	0.031	0.06^32^
Fluconazole	4->16	4->16	4-8^33,34^
Voriconazole	0.008	0.03	.002^33^
Anidulafungin	> 16	> 16	> 8^35^
Caspofungin	> 16	> 16	> 16^36^
Micafungin	> 16	> 16	> 16^37^
Amphotericin B	0.125, 0.016^#^	0.25, 0.5^#^	1^34^
5-Flucytosine	0.25–0.5	0.125	0.125-8*^38^

Most of the antifungals tested against *C. albicans* and *C. neoformans* were within the range of expected values, obtained from the literature, in both medias. An exception was caspofungin which, due to its lipophilic chemical structure, had better activity in NBS plates than PS plates (literature values are primarily in PS plates). We have previously described the effect of NBS plates on the antibacterial MIC of some antibiotics^23^. Furthermore, the CLSI M60: Performance standards for Antifungal Susceptibility Testing of Yeasts reports significant variability in results of in vitro testing of caspofungin MIC between laboratories^24^. The MIC of ketoconazole against *C. neoformans* ATCC 208821 H99 in YNB were higher than the reference MIC. Other papers have suggested the same outcome, in which higher ketoconazole MIC results were observed against *C. neoformans*^25,26^. An additional MIC was done using the same methodology with PS plates and YNB media to validate NBS results and also chosen conditions (Appendix Tables 4 and 6 ). Comparison of MIC results is limited due to the lack of literature data available for the two strains used. The CLSI M60 Performance standards only provides MIC values for amphotericin B, fluconazole, and 5-flucytosine, against *C. albicans* ATCC 90028. Further, the CLSI MIC values were determined using the broth macrodilution method, rather than the broth microdilution method. Since 5-flucytosine is a prodrug, used in combination with amphotericin B for treatment of cryptococcosis, there are limited reference MIC values for the drug alone against *C. neoformans*^27^.

### High throughput screening performance

 As part of the Community for Antimicrobial Drug Discovery (COADD)^39,40^, we tested a library of more than 300,000 compounds for antifungal activity, using these optimised assay conditions. The assay has been performed on over 5,500 NBS 384-well plates. The assay against *C. albicans* had a very good and consistent assay performance, with an average Z’-Factor of 0.94 (Stdev = 0.08; 2862 plates) and a rejection rate of 2.2% (64 plates) due to low Z’-Factor’s (< 0.25) or inconsistency in automatic dispensing of either compound solution or fungal broth. For the assay against *C. neoformans* the performance was slightly lower, displaying more variations, with an average Z’-Factor of 0.76 (Stdev = 0.16; 2701 plates), however with similar low rejection rate of 2.8%.(79 plates).

## Discussion

### Incubation time

**Fig. 2:**
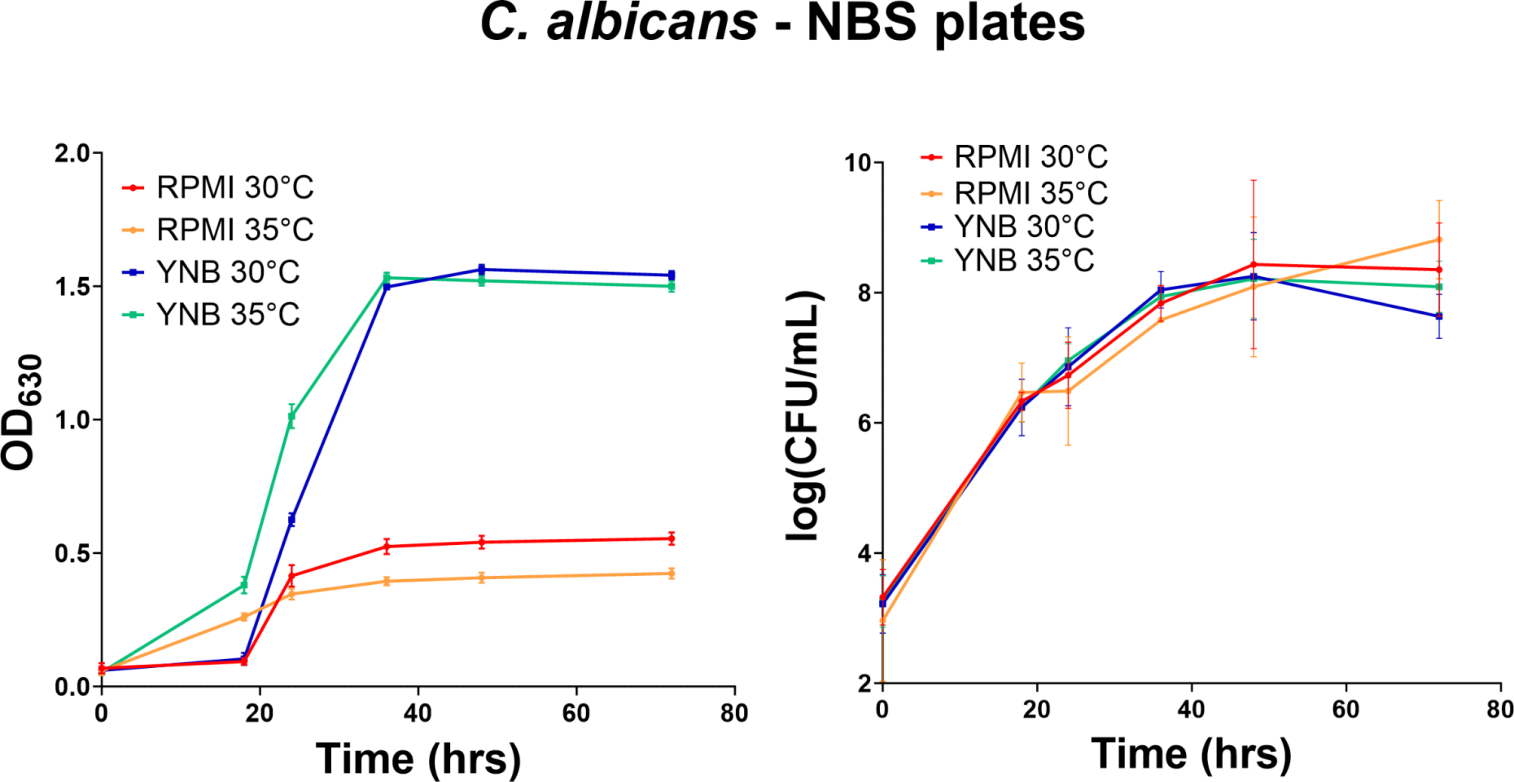
Growth curve measured as OD_630_ (*n = 4*) and by log(CFU/mL) (*n* = 2)for *C. albicans* using RPMI and YNB media at 30 °C and 35 °C in NBS 384-well plates.

 The CLSI M27 guidelines recommend taking note of the difference in end point readings, since the difference between readings at 24 and 48 h may be more clinically relevant for some *Candida* strains, hence this study tested 24, 36 and 48 h incubation for *C. albicans.* In our experiments, there was no notable difference in the MIC values between these incubation periods. However, the extent of the OD_630_ values increased notably from 24 to 36 h while remaining constant for the period from 36 to 48 h, with only small increase in the CFU/mL counts (Fig. 2). Based on these observations, we selected the 36 h time point, close to the end of the exponential phase, as the optimum incubation period for *C. albicans.*

**Fig. 3:**
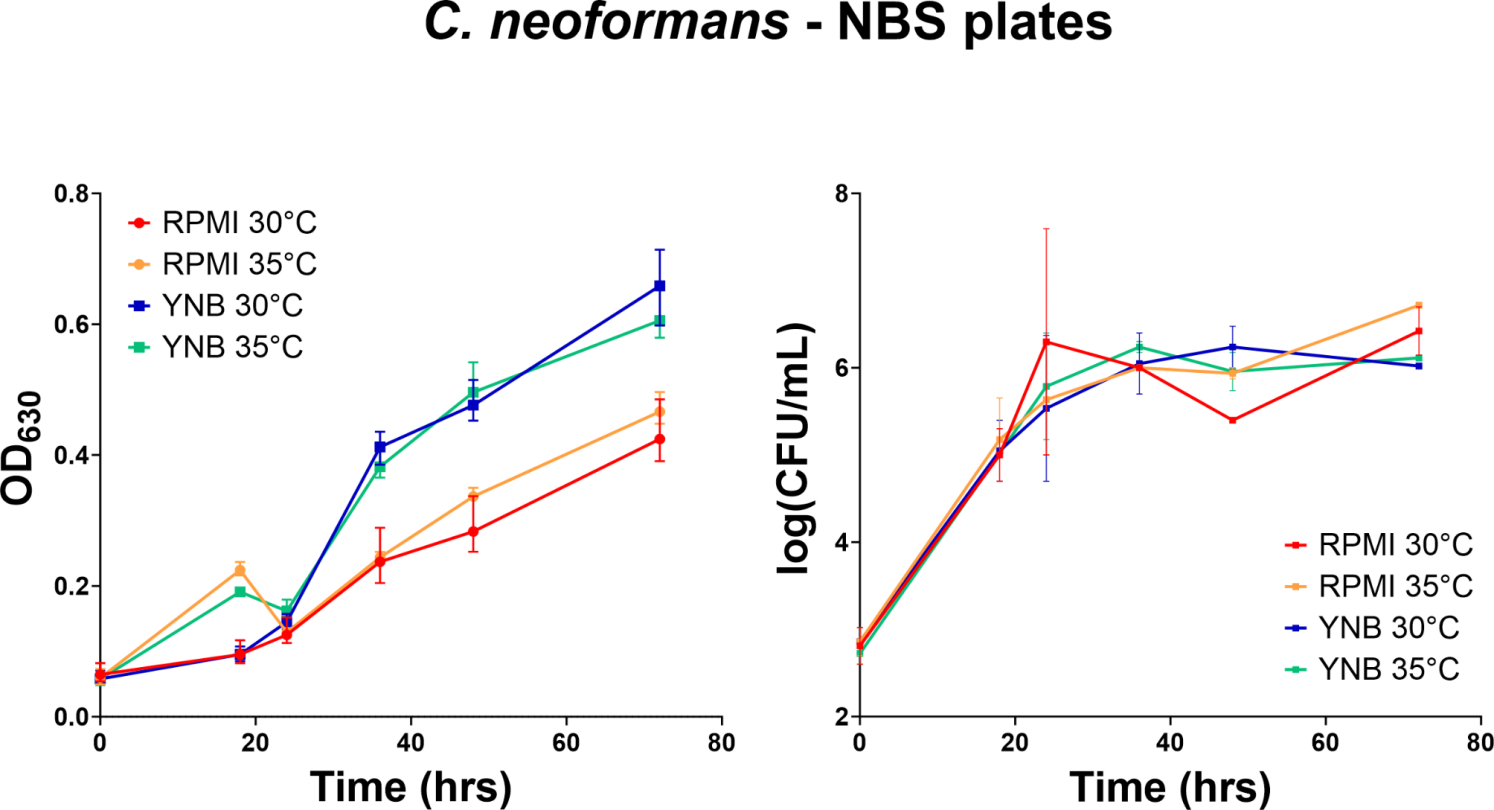
Growth curve measured as OD_630_ (*n =* 4) and by log(CFU/mL) (*n* = 2) for *C. neoformans* using RPMI and YNB media at 30 °C and 35 °C in NBS 384-well plates.

Figure 3 shows that *C. neoformans* is in exponential phase even after 72 h of incubation according to the OD_630_ readout, even though the CFU/mL counts do not differ significantly after 36 h incubation time. Visual inspection of the 384-well plates after 72 h incubation indicated a reduction of the liquid media by around 50% through evaporation. Based on these results, 36 h incubation time was chosen for high throughput antifungal screening, as this showed an acceptable evaporative media loss of around 15%, but still produced a large enough difference in OD between positive and negative controls to determine accurate inhibition values for single concentration primary screening.

### Media

 The results for *C. albicans* (Table 2) high throughput antifungal screening showed a comparable range of MIC values using all tested medias, however YNB had more consistent values across all replicates. Additionally, YNB had significant higher OD_630_ readouts, providing a larger difference between positive and negative controls and more statistical significance to the calculation of inhibition .

*C. neoformans* also had significantly increased OD_630_ readings (Fig. 3) and more reproducible MIC results with YNB when compared with RPMI (Table 1), and with both 80% and 50% inhibition-cutoff MIC analysis remaining constant in YNB (Appendix Table 5). Meanwhile, the MIC analysis using 80% or 50% inhibition-cutoff varied approximately two to three fold in RPMI, with both *C. neoformans* and *C. albicans*, due to a more gradual dose response curve in RPMI, compared to more steeper dose response in YNB. However, 80% inhibition-cutoff MIC values across all media types and for the majority of tested antifungals were within the ranges currently presented in the literature. Based on these observations, we selected YNB media as the optimum media for both *C. albicans* and *C. neoformans.*

### Endpoint readout

 The addition of resazurin to *C. neoformans* plates before reading was included to aid the accuracy of the calculated optical density separation between positive and negative controls. Although *C. neoformans* has a significantly higher OD when grown in YNB compared to RPMI, the OD_630_ measurement at the 36 h time point was too low to provide a highly significant separation between positive and negative controls (Z’-factors > 0.5). This is especially important for primary screening at single compound concentrations, which relies on good Z’-factors to select actives for further dose response confirmation. To maintain the 36 h incubation time chosen for its advantages in limiting evaporative loss and edge effect, resazurin was added to the 384-well plates and growth inhibition measured by calculating the difference in absorbance between 570 and 600 nm. The addition of resazurin was done by bulk dispensers or liquid handling robots, with reading the plates after a short incubation time. This adds only little resources and time to the high-throughput screening workflow for *C. neoformans* while simultaneously improving the quality control of each screen.

In summary, this study concludes that YNB media, incubated at 35 °C for 36 h, in 384 well-NBS plates are the optimal conditions to run high throughput antifungal screening for non-fermentative yeast like *C. albicans* and *C. neoformans*, using either OD_630_ or OD_570 − 600_ with the addition of resazurin. Using these validated conditions we have been able to test over 300,000 compounds against these two yeast species, while also testing selected compounds against a range of different *Candida* and *Cryptococcus* species and clinical isolates with similar assay performances.

While the assay conditions of using YNB media and NBS plates could be applied to other yeast or filamentous fungi, the incubation time may need to be optimised to provide an absorbance readout suitable for high throughput screening, consistent across a 384-well plate format and with statistically significant separation between growth and no growth.

## Methods

### Strains and antifungals

 Strains tested were purchased from the ATCC collection, *Candida albicans* (Robin) ATCC^®^ 90028, *Cryptococcus neoformans var. grubii* (H99) ATCC^®^ 208821. The species were confirmed using the VITEK 2 Compact system and YST identification card. The strains *C. albicans* and *C. neoformans* were cultured for 24 and 48 h, respectively, from a fresh glycerol stock onto YPD agar at 35 °C. The strains were then maintained on YPD agar and kept at 4 °C before being used for inoculum preparation for the assays.

Fluconazole (Sigma Aldrich Cat No. 8929), amphotericin B (Sigma Aldrich Cat No. A4888 as powder; Sigma Aldrich Cat No. A2942 as solution in deionized water), 5-fluorocytosine (Sigma Aldrich Cat No. F7129), posaconazole (Sigma Aldrich Cat No. 32103), voriconazole (Cayman Chemical Cat No. 15633), ketoconazole (Sigma Aldrich Cat No. K1003), itraconazole (Sigma Aldrich Cat No. I6657), caspofungin diacetate (Sigma Aldrich No. SML0425), anidulafungin (Carbosynth Cat No. FA16270) and micafungin sodium (Carbonsynth Cat No. FM34148) were purchased from the indicated suppliers. All antifungals were prepared fresh, at 1.28 mg/mL in 100% dimethyl sulfoxide (DMSO) from powder stocks. The exception was amphotericin B, in which both the powder form and solution stock of 250 µg/mL were included.

### Growth curve assay

 Colonies from each culture were re-suspended in 5 mL of sterile water and adjusted to a density of OD = 0.3–0.4 at 530nm (OD_530_) wavelength, resulting in a yeast stock suspension of 10^7^ cells per mL. These stock suspensions were diluted to the final concentration of 2.5 × 10^3^ CFU/mL in fresh YNB broth (Becton Dickinson Cat No. 233520) supplemented with 2% glucose, or RPMI1640 culture medium (Sigma Cat No. R6504-10 × 1 L) with the addition of 0.165 mol/L MOPS buffer.The RPMI-1640 media was adjusted to a pH of 7, with the addition of 1 mol/L sodium hydroxide, according to CLSI guidelines.

48 µL of the stock suspensions were dispensed, using an automated 16 channel pipette, into each well of the compound containing NBS 384-well plates (Corning Cat No. 3640) and PS 384-well plates (CorningCat No. 3680) in duplicate (*n* = 2). Fresh media was used as a negative control in the last column. Plates were covered and incubated at 30 °C and 35 °C.

Growth of *C. albicans* and *C. neoformans* was determined measuring absorbance at OD_630_, at 0, 18, 24, 36, 48 and 72 h time points, measured using a Biotek Synergy HTX plate reader. Growth curves were generated using an average of the OD_630_ values across all corresponding growth wells on each plates (*n* = 176 wells on 384-well plates with two media conditions and two columns of media only controls). Upon inoculation of the 384-well growth curve plates, a 20 µL sample from each yeast stock suspension was taken and streaked out on YPD agar plates to measure a baseline CFU/mL of the 0 h time point. Subsequently, at each following time point, 20 µL of each strain was taken from the 384-well growth plates for all conditions. The *C. albicans* culture was diluted 1:10000 for time points 18 h, 24 h and 36 h, and 1:100000 for time points 48 h and 72 h. The *C. neoformans* culture was diluted 1:1000 for all time points 18 h to 72 h. The samples were struck out on YPD agar and incubated at 35 °C, and after 36 h the colonies were counted to determine the CFU/mL for each condition.

### High throughput antifungal screening

 Based on the results of the growth curve and subsequent literature search, only specific time points were chosen for each strain to compare MIC results and validate an optimal high throughput screening method for both *C. albicans* and *C. neoformans*.

For *C. albicans*, we chose to compare microbroth dilution using YNB media incubated at 35 °C for 36 h in 384 well plates versus using RPMI-1640 incubated at 35 °C for 48 h, as specified in the CLSI guidelines. For *C. neoformans*, we chose to compare microbroth dilution using YNB media incubated at 35 °C for 36 h in 384 well plates versus using RPMI-1640 incubated at 35 °C for 72 h, as specified in the CLSI guidelines. Fungal strains and subsequent fungi suspension were prepared in the same manner as the growth curve.

The antifungal standards were added to a 384-well deep well polypropylene V-bottom plate (PerkinElmer Cat No. 6008690), in duplicate, at 10 times the final test concentration. The compounds were then serially diluted two-fold in 20% DMSO, 16 times down the wells of the plate. The Biomek liquid handling robot was used to stamp 5 µL from each well of the 384 well deep well polypropylene V-bottom plate, containing the antifungal standards, into NBS  384 well plates (Corning Cat No.3640) and PS 384-well plates (Corning Cat No. 3680). Then, 45 µL of the fungi suspension in each media was added to the compound-containing 384 well plates, giving a final cell concentration of 2.5 × 10^3^ CFU/mL and a final compound concentration range from  16 µg/mL to 0.00049 µg/mL. All plates were covered with lids and incubated under the described conditions, without shaking.

All tested antifungals had a final DMSO concentration of 2% or lower. For all plates, a DMSO control was added to ensure inhibition was not caused by DMSO concentration. All plates included a positive and negative growth control column in order to calculate inhibition percentage using absorbance.

Growth inhibition of *C. albicans* was determined by measuring absorbance at OD_630_, while the growth inhibition of *C. neoformans* was determined measuring the difference in absorbance OD_570 − 600_, after the addition of resazurin (0.001% final concentration) and a 5 min short incubation time. All absorbance values were obtained using a Biotek Synergy HTX plate reader. In both cases, the percentage of growth inhibition was calculated for each well, using the negative growth control (media only) and positive growth control (fungi without inhibitors) on the same plate. The MIC was determined as the lowest concentration at which growth was fully inhibited, defined by an inhibition ≥ 80% and ≥ 50%.

## Electronic supplementary material

Below is the link to the electronic supplementary material.

Supplementary Material 1

## Data Availability

All relevant data are available in the Supplementary Material or from the corresponding author upon request to the following email: j.zuegg@uq.edu.au.
