# Supplementary material for: Standardisation of high throughput microdilution antifungal susceptibility testing for Candida albicans and Cryptococcus neoformans
Source: Sci Rep. 2024 Oct 8;14:23407. doi: 10.1038/s41598-024-74068-2 (PMC11461513; doi:10.1038/s41598-024-74068-2)
Supplement: Supplementary file 1 — Supplementary Material 1 [file 41598_2024_74068_MOESM1_ESM.pdf]

Standardisation of High throughput microdilution antifungal susceptibility testing for *Candida albicans* and *Cryptococcus neoformans*

Holly Floyd[1#], Angela M. Kavanagh[1#], Gabrielle J. Lowe[1#], Maite Amado[1], James A. Fraser[2], Mark A. T. Blaskovich[1], Alysha G. Elliott[1], Johannes Zuegg[1*]

[1] Community for Open Antimicrobial Drug Discovery, Centre for Superbug Solutions, Institute for Molecular Bioscience, University of Queensland, Qld, Australia.

[2]School of Chemistry and Molecular Biosciences, University of Queensland, Qld, Australia.

#These authors contributed equally to this work.

*To whom correspondence should be addressed. E-mail: j.zuegg@uq.edu.au

Supplementary appendix

Appendix Formula 1: Edge Effect as percentage growth

$$Edge\ Effect\ (\%) = 100 * \left(\frac{mean\left(OD_{600}{}^{Edge\ wells}\right)}{mean\left(OD_{600}{}^{Non-Edge\ wells}\right)} - 1\right)$$

Appendix Formula 2: Edge Effect as Z-Score

$$ZScore_{Edge\ Effect} = \frac{\left|mean\left(OD_{600}{}^{Edge\ wells}\right) - mean\left(OD_{600}{}^{Non-Edge\ wells}\right)\right|}{stdev\left(OD_{600}{}^{Non-Edge\ wells}\right)}$$

Appendix Formula 3: Z'-Factor

$$Z' - Factor = 1 - \frac{3\left(MAD_{Positive\ control} + MAD_{Negative\ control}\right)}{\left|median_{Positive\ control} - median_{Negative\ control}\right|}$$

Appendix Figure 1: Growth curve measured as OD_{630} (*n*=*4*) and log CFU/mL (*n*=*2*) for *C. albicans* using RPMI and YNB media at 30 °C and 35 °C in 384 well PS plates.

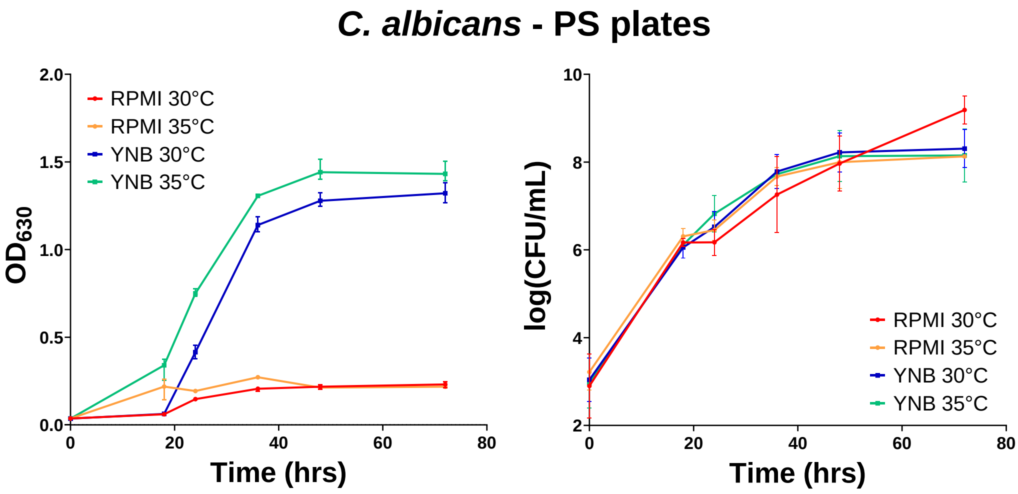

Appendix Figure 2: Growth curve measured as OD_{630} (*n*=*4*) and log CFU/mL (*n*=*2*) for *C. neoformans* using RPMI and YNB media at 30 °C and 35 °C in 384 well PS plates.

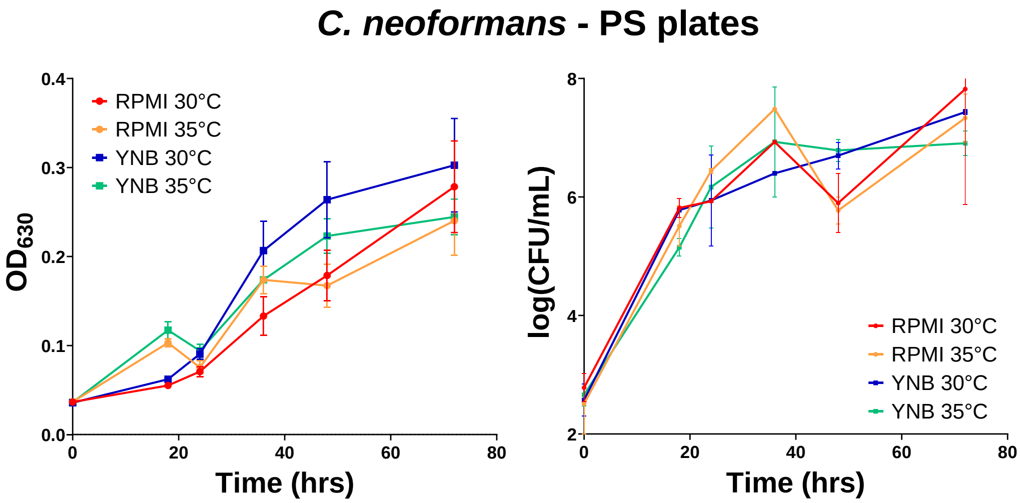

Appendix Table 1: Edge effect in growth of *C. albicans* ATCC 90028 in 384-well, measured as OD_{630}, calculated as average percentage between edge well and wells in the middle, and as Z-Score of edge wells against the population of wells in the middle of the plate.

***Candida albicans* ATCC 90028**									
**Plate Type**	**Time (hrs)**	**Edge Effect (%)**				**Z-Score**			
		**RPMI**		**YNB**		**RPMI**		**YNB**	
		**30 °C**	**35 °C**	**30 °C**	**35 °C**	**30 °C**	**35 °C**	**30 °C**	**35 °C**
**NBS**	18	-10.2	-10.9	-23.3	-22.0	0.77	0.62	2.44	0.99
	24	-6.9	3.7	0.5	-5.8	0.33	0.23	0.02	0.18
	36	-4.8	2.5	-2.0	-1.1	0.29	0.19	0.06	0.03
	48	-2.6	6.0	-1.2	0.3	0.17	0.48	0.04	0.01
	72	-0.6	7.5	-1.0	0.6	0.04	0.65	0.03	0.02
**PS**	18	18.1	-17.7	1.4	-7.7	0.26	0.42	0.28	0.03
	24	48.4	17.5	13.0	12.1	0.29	0.02	0.14	0.03
	36	43.2	-6.4	9.6	2.9	0.64	0.03	0.31	0.25
	48	37.4	22.2	8.9	6.3	1.49	0.20	0.69	0.39
	72	46.2	25.4	10.2	6.9	0.92	0.62	0.86	0.47

Appendix Table 2: Edge effect in growth of *C. neoformans* ATCC 208821 in 384-well, measured as OD_{630}, calculated as average percentage between edge well and wells in the middle, and as Z-Score of edge wells against the population of wells in the middle of the plate.

Cryptococcus neoformans ATCC 208821									
**Plate Type**	**Time (hrs)**	**Edge Effect (%)**				**Z-Score**			
		**RPMI**		**YNB**		**RPMI**		**YNB**	
		**30° C**	**35° C**	**30° C**	**35° C**	**30° C**	**35° C**	**30° C**	**35° C**
**NBS**	18	3.6	-8.5	4.1	0.6	0.26	0.42	0.28	0.03
	24	4.4	-0.3	2.6	0.6	0.29	0.02	0.14	0.03
	36	7.9	0.3	-5.7	5.5	0.64	0.03	0.31	0.25
	48	17.3	-2.1	13.3	7.4	1.49	0.20	0.69	0.39
	72	7.9	-4.9	15.5	7.9	0.92	0.62	0.86	0.47
**PS**	18	20.7	30.9	20.4	8.2	1.63	1.20	1.85	0.48
	24	35.5	50.3	37.3	36.4	2.13	3.38	2.24	2.79
	36	57.2	64.4	70.2	55.5	2.50	3.71	2.14	2.96
	48	60.7	74.4	64.9	58.1	2.56	4.12	2.22	3.49
	72	69.1	75.8	66.0	57.3	3.75	5.56	2.91	2.58

Appendix Table 3: MIC_2 (μg/mL) or CLSI score 2 of tested antifungals against *C. albicans* ATCC 90028 in 384 well non-binding surface (NBS) plates, by OD_{630} readout (*n=4*).

**Antifungal**	Inhibition of ***C. albicans*** **ATCC 90028** using **NBS** plates **MIC_2 (μg/mL)** using 50% inhibition		
	**RPMI 48 hr**	**YNB 36 hr**	**CLSI or Literature**
**Ketoconazole**	0.0625-0.25	0.031	0.031[1]
**Posaconazole**	0.004	0.002	-
**Itraconazole**	0.0019	0.031	0.016[1]
**Fluconazole**	0.25	0.5	0.25-1[2]
**Voriconazole**	0.004-0.008	0.002	0.004-0.016[3]
**Anidulafungin**	0.0156	0.002	0.03
**Caspofungin**	0.00097	0.0005	0.125[4]
**Micafungin**	0.0078-0.0156	≤ 0.0004	0.016[5]
**Amphotericin B**	0.5	≤ 0.0002	0.5-2[2]
**5-Flucytosine**	0.0625-0.125	0.0625	0.5-2[2]

Appendix Table 4: MIC (μg/mL) of tested antifungals against *C. albicans* ATCC 90028 in 384-well Polystyrene (PS) plates, by OD_{630} readout (*n=4*).

**Antifungal**	Inhibition of ***C. albicans*** **ATCC 90028** using **PS** plates **MIC (μg/mL)**		
	**RPMI 48 hr**	**YNB 36 hr**	**CLSI or Literature**
**Ketoconazole**	0.25-0.125	0.25-0.125	0.031[1]
**Posaconazole**	0.125-0.03	0.125-0.03	-
**Itraconazole**	0.125-0.06	0.125-0.06	0.016[1]
**Fluconazole**	0.5	0.5	0.25-1[2]
**Voriconazole**	0.015-0.008	0.015-0.008	0.004-0.016[3]
**Anidulafungin**	0.25-0.03	0.25-0.03	0.03
**Caspofungin**	0.25-0.125	0.25-0.06	0.125[4]
**Micafungin**	0.25-0.06	0.0019-0.0004	0.016[5]
**Amphotericin B**	8	8	0.5-2[2]
**5-Flucytosine**	0.125	0.125	0.5-2[2]

Appendix Table 5: MIC_2 (µg/mL) or CLSI score 2 of tested antifungals against *C. neoformans* var. grubii (H99) ATCC 208821 in 384-well non-binding surface plates, by $OD_{570-600}$ readout with resazurin (*n=4*).

Antifungal	Inhibition of ***C. neoformans ATCC 208821*** using **NBS** plates **MIC_2 (µg/mL)** using 50% inhibition		
	**RPMI 48 hr**	**YNB 36 hr**	**CLSI or Literature**
**Ketoconazole**	1	0.008	0.06[6]
**Posaconazole**	0.016-0.031	0.008	0.063[7]
**Itraconazole**	0.016-0.031	0.008	0.06[6]
**Fluconazole**	8	2	4-8[7,8]
**Voriconazole**	0.062-0.125	0.031	.002[7]
**Anidulafungin**	>12.8	>12.8	>8[9]
**Caspofungin**	2-4	4	>16[10]
**Micafungin**	>1024	>1024	>16[11]
**Amphotericin B**	0.5	0.062-0.125	1[8]
**5-Flucytosine**	0.0625	1-2	0.125-8*[12]

Appendix Table 6: MIC (µg/mL) of tested antifungals against *C. neoformans* var. *grubii* (H99) ATCC 208821 in 384 well Polystyrene (PS) plates, by $OD_{570-600}$ readout with resazurin (*n=4*).

Antifungal	Inhibition of ***C. neoformans ATCC 208821*** using **PS** plates **MIC (µg/mL)**		
	**RPMI 48 hr**	**YNB 36 hr**	**CLSI or Literature**
**Ketoconazole**	2	2-1	0.06[6]
**Posaconazole**	1-4	1-4	0.063[7]
**Itraconazole**	2	2	0.06[6]
**Fluconazole**	8	8	4-8[7,8]
**Voriconazole**	0.125	0.125-0.06	.002[7]
**Anidulafungin**	>16	>16	>8[9]
**Caspofungin**	>512	>512	>16[10]
**Micafungin**	>8	>8	>16[11]
**Amphotericin B**	8	8	1[8]
**5-Flucytosine**	0.25-0.125	0.25-0.06	0.125-8*[12]

References

1 Nyilasi, I. *et al.* In vitro synergistic interactions of the effects of various statins and azoles against some clinically important fungi. *FEMS Microbiol Lett* **307**, 175-184 (2010). https://doi.org:10.1111/j.1574-6968.2010.01972.x

2 CLSI. Performance Standards for Antifungal Susceptibility Testing of Yeasts. 1st ed. CLSI supplement M60. (2017).

3 Mallie, M. *et al.* In vitro susceptibility testing of Candida and Aspergillus spp. to voriconazole and other antifungal agents using Etest: results of a French multicentre study. *Int J Antimicrob Agents* **25**, 321-328 (2005). https://doi.org:10.1016/j.ijantimicag.2004.11.010

4 Wiederhold, N. P., Najvar, L. K., Bocanegra, R. A., Kirkpatrick, W. R. & Patterson, T. F. Caspofungin dose escalation for invasive candidiasis due to resistant *Candida albicans*. *Antimicrob Agents Chemother* **55**, 3254-3260 (2011). https://doi.org:10.1128/AAC.01750-10

5 Nussbaumer-Proll, A., Matzneller, P., Eberl, S. & Zeitlinger, M. Pulmonary surfactant impacts in vitro activity of selected antifungal drugs against *Candida krusei* and *Candida albicans*. *Eur J Clin Microbiol Infect Dis* (2024). https://doi.org:10.1007/s10096-024-04799-7

6 Keerativasee, S. *et al.* Heteroresistance to fluconazole among isolates of *Cryptococcus neoformans* in Northern Thailand. *Afr J Microbiol Res* **7**, 4096-4102 (2013). https://doi.org:https://doi.org/10.5897/AJMR2013.5958

7 Gast, C. E., Basso, L. R., Jr., Bruzual, I. & Wong, B. Azole resistance in *Cryptococcus gattii* from the Pacific Northwest: Investigation of the role of ERG11. *Antimicrob Agents Chemother* **57**, 5478-5485 (2013). https://doi.org:10.1128/AAC.02287-12

8 Lu, R. Y. *et al.* New Triazole NT-a9 Has Potent Antifungal Efficacy against *Cryptococcus neoformans* In Vitro and In Vivo. *Antimicrob Agents Chemother* **64** (2020). https://doi.org:10.1128/AAC.01628-19

9 Ding, H. *et al.* BG40018: a promising drug candidate for the treatment of invasive fungal infections. *Int J Clin Exp Med* **10**, 14401-14407 (2017).

10 Pianalto, K. M., Billmyre, R. B., Telzrow, C. L. & Alspaugh, J. A. Roles for Stress Response and Cell Wall Biosynthesis Pathways in Caspofungin Tolerance in *Cryptococcus neoformans*. *Genetics* **213**, 213-227 (2019). https://doi.org:10.1534/genetics.119.302290

11 Xu, Y. *et al.* Spiro[benzoxazine-piperidin]-one derivatives as chitin synthase inhibitors and antifungal agents: Design, synthesis and biological evaluation. *Eur J Med Chem* **243**, 114723 (2022). https://doi.org:10.1016/j.ejmech.2022.114723

12 Li, M., Liao, Y., Chen, M., Pan, W. & Weng, L. Antifungal susceptibilities of Cryptococcus species complex isolates from AIDS and non-AIDS patients in Southeast China. *Braz J Infect Dis* **16**, 175-179 (2012). https://doi.org:10.1016/s1413-8670(12)70301-x
